# An Audit Cycle Evaluating Timeliness of Post-Discharge Follow-Up for Patients Discharged From an Adult General Psychiatry Inpatient Ward in Wales

**DOI:** 10.1192/bjo.2026.11672

**Published:** 2026-06-30

**Authors:** Hui Rou Lim, Emine Demiral

**Affiliations:** Hywel Dda Health Board, Llanelli, United Kingdom

## Abstract

**Aims:**

This audit cycle evaluated adherence to recommended follow-up timeframes (NICE guidance/ RCPsych core standards) for patients discharged from a general adult psychiatry ward in Wales.

**Methods:**

Cycle 1 assessed compliance with NICE guidance recommending review within 7 days for all patients and within 48 hours for those at risk of suicide.This included all patients discharged from Bryngofal ward between January to March 2024. Suicide risk was determined using the Wales Applied Risk Research Network (WAARN) formulation at discharge. The first scheduled post-discharge care contact was recorded.

Following staff education and clarification of local accepted standards, cycle 2 assessed compliance with the RCPsych core standards of review within 3 days for all patients. The audit was repeated for all discharges between November 2024 to January 2025 using the RCPsych standards.

**Results:**

Cycle 1: Of 44 patients, 42 (95%) received follow-up within NICE-recommended timeframes. Instances of non-compliance were related to transfer of care to another health board and incomplete discharge risk documentation. Interventions included reinforcement of discharge risk documentation and clarification of local follow-up standards. Based on evidence from the National Confidential Inquiry into Suicide and Safety in Mental Health (days 2–3 post-discharge is a high-risk period), our local health board aligned with the RCPsych core standards recommending review within 3 days.

Cycle 2: Of 19 patients, 18 (95%) received follow-up within 3 days. The single delayed review occurred due to a communication lapse between inpatient and community teams.Improved communication between ward and community teams was emphasised during ward round to support timely review.

**Conclusion:**

Compliance with national standards for timely follow-up was high and sustained across both cycles. The findings highlight the critical role of effective communication and clear recording between inpatient teams, community mental health teams, and care coordinators to ensure timely follow-up and patient safety. Simple measures, including staff education, clear discharge risk documentation, enhanced multidisciplinary communication and clarification of local standards can support ongoing high quality follow-up care.

